# Waterpipe Smoking among Bladder Cancer Patients: A Cross-Sectional Study of Lebanese and Jordanian Populations

**DOI:** 10.1155/2021/6615832

**Published:** 2021-04-19

**Authors:** Elio Jabra, Amal Al-Omari, Fadi Haddadin, Walid Alam, Khawlah Ammar, Maya Charafeddine, Mohammad Alrawashdeh, Nour Kasasbeh, Charbel Habis, Deborah Mukherji, Sally Temraz, Ali Shamseddine

**Affiliations:** ^1^Division of Hematology and Oncology, Department of Internal Medicine, American University of Beirut Medical Center (AUBMC), Beirut, Lebanon; ^2^Office of Scientific Affairs and Research, King Hussein Cancer Center (KHCC), Amman, Jordan

## Abstract

**Background:**

Bladder cancer (BC) is the second most reported cancer in Lebanon and the fifth in Jordan. Its risk factors are mainly smoking and occupational exposure to aromatic amines. In these countries where smoking and bladder cancer are highly prevalent, the role of waterpipe smoking (WPS) in bladder cancer is less investigated. We aim to compare two sets of patients between Lebanon and Jordan, focusing on their smoking habits, WP use, occupational exposure, and the grade/invasiveness of their bladder cancer.

**Methods:**

This is a cross-sectional study that compares the smoking culture between two sets of populations with bladder cancer, from two different countries. We recruited 274 bladder cancer patients over the 18 years of age at the American University of Beirut Medical Center (AUBMC), and 158 bladder cancer patients over the age of 18 years at the King Hussein Cancer Center (KHCC).

**Results:**

7.7% of Lebanese patients had significantly more positive family history of bladder cancer compared to 13.9% of Jordanian patients (*p* = 0.045). Another significant finding is that the majority of Lebanese patients 70.7% reported being frequently exposed to secondhand smoking, mainly cigarettes, versus only 48.6% of Jordanian patients (*p* < 0.001). The increasing smoking trend among Lebanese females is remarkably the highest in the region, which contributed to the overall increase in smoking rates in the country. 17.1% of the Lebanese smoking patients are mainly but not exclusively WP smokers of which 6.3% are daily WP smokers, similarly 17.1% of the Jordanian patients of which 3.2% are daily WP smokers. There were 71.5% of Lebanese patients who had a noninvasive BC versus 40% of Jordanian patients (*p* < 0.001), and more than one-third reported an occupational exposure to one of the risk factors of BC in both groups.

**Conclusions:**

Bladder cancer incidence is on the rise in both Jordan and Lebanon along with different smoking types. It is necessary to impose prevention policies to prevent and control the high smoking prevalence. Bladder cancer invasiveness is higher in Jordan compared to universal data.

## 1. Introduction

Bladder cancer (BC) is the 9^th^ most common cancer and the 14^th^ leading cause of death due to cancer worldwide. The highest rates are reported in Europe, North America, Australia, and Egypt, in comparison to the relatively low rates in the Far Eastern countries [1].

Histologically, most cases of BC are transitional cell carcinomas (TCC) (90%); rare are squamous cell carcinoma (3-5%), adenocarcinoma (0.5 to 2%), small cell carcinoma (less than 0.5%), and sarcoma, sarcomatoid tumors, paraganglioma, melanoma, and lymphoma (less than 0.1%) [2].

Risk factors associated with bladder cancer are as follows: smoking (cigarettes), noncigarette smoking (cigar, pipe, WP, and smokeless tobacco; less investigated), arsenic in drinking water (concentration higher than 300 *μ*g/l), arsenic exposure (in air, food, and occupational hazards), occupational exposure to aromatic amines (2-naphthylamine, 4-aminobiphenyl, and benzidine), and 4,4′-methylenebis (2-chloroaniline). The last two risk factors can be found in the chemical, dye and rubber industries, in hair dyes, paints, fungicides, cigarette smoke, plastics, metal, and motor vehicle exhaust [3]. We note that it may take up to several years or decades between exposure and the onset of the subsequent cancer.

Waterpipe smoking (WPS, also known as hookah, shisha, nargileh, and hubble-bubble) has an estimated prevalence of 100 million worldwide, mostly among adolescents of the Middle East [4, 5] A Lebanon-based longitudinal study including 228 exclusive WPS adolescent smokers revealed that nicotine dependence among WPS was associated with initiating smoking at a younger age and believing that WP smokers have more friends among other factors [6]. In addition, its affordability and the large number of flavor options transformed this smoking habit into a popular social activity among youngsters in the Middle East [7]. A study involving five Palestinian universities revealed that WPS was 24.4% among students which surpassed the 18% for cigarette smoking. While it remains more common in males, the gender-gap is smaller than that of cigarette smoking [8]. A cross-sectional survey conducted among 181 self-reported WP smokers in shisha cafes in Qatar showed that participants viewed WPS as a safer alternative to cigarettes and had no problem having their children involved in WPS [9]. However, the previous misconceptions about the safety and reduced harm of WPS have been disproved [10]. Recent research revealed the presence of multiple dangerous toxins and carcinogens in its smoke and the lack of their filtration through the water. In addition, a study examining the underlying risks of subchronic WP exposure in mice showed increased urea serum levels, lungs, kidneys, bone marrow, and liver damage compared to controls [11]. In previous studies, a significant association of WPS with lung cancer was established; gastric and esophageal cancers were observed to have weak associations with WPS [12].

Lebanon, a middle-income country in the Middle East, was found to have one of the highest estimated age-standardized incidence rates of bladder cancer worldwide [13]. While bladder cancer ranks among the top 5 cancers in the West, BC in Lebanon is the third most common cancer as reported from 2005 to 2011 [13]. It is the second most reported cancer [14] in Lebanese males and fourth most common cancer in males reported in Jordan [14], with incidence increasing with age, rarely occurring before the age of 40–50 and arising most frequently in the 7^th^ decade of life [15]. The incidence rates of bladder cancer in Lebanese males are nearly double the rates reported in neighboring countries and almost triple that of Jordan, with the incidence rate reaching 34 cases per 100,000 [14], which triggered interest in its possible etiology considering their lifestyle.

Tobacco smoke is the most recognized risk factor for both TCC and non-TCC BC [16], and the prevalence of cigarette smoking in Lebanon is among the highest recorded in the region [17]. Data from the WHO global report on trends in prevalence of tobacco showed that age-standardized prevalence among people aged over 15 was 42.6% in Lebanon [18].

In Jordan, tobacco smoking is popular among the general population. A study done in 2019 in collaboration with the World Health Organization showed that the prevalence of tobacco smoking was 66% in men and 17% in women [19, 20]. Among the smokers, 93% used cigarette smoking and 8.6% used WPS. A study involving WP smokers from Jordan, Egypt, and Palestine showed that those who are male, in the 22-29 age category, unemployed, and started smoking at a younger age smoked other types too. Up to 45% of the Jordanian WP smokers smoked at least one additional tobacco product [21]. Another study of 500 women showed that 82.4% and 32.8% were exposed to secondhand cigarette and WPS, respectively, in their houses and public places [22]. While Jordan has smoking-free policies, they are poorly enforced leading to excessive exposure in both public and private areas [23].

Data associating WPS to bladder cancer is scarce globally and limited to literature reviews and some case controls. Zheng et al. found that those who ever smoked a WP, but did not smoke cigarettes, had a borderline significant association with an increased risk of urothelial carcinoma (OR = 1.3 and 95% CI [1.0-1.8]) compared to nonsmokers. There was no association observed between squamous cell carcinoma and WPS [24]. Findings from a systematic review exploring the relationship between waterpipe smoking and health outcomes reported a pooled OR = 1.25 and 95% CI 0.99-1.57 [25].

Considering the scarcity of data on risk factors related to the high incidence of bladder cancer in the region, we conducted a pilot study at the American University of Beirut Medical Center (AUBMC) and King Hussein Cancer Center (KHCC). Our study is aimed at comparing smoking rates and other risk factors linked to BC, while also looking at BC grade and invasiveness between Lebanon and Jordan. We hope that this paper will highlight the importance of implementing prevention policies and raise awareness on the dangers of tobacco smoking, WPS, and secondhand smoking.

## 2. Materials and Methods

### 2.1. Study Design and Subjects

Our study identified a group of bladder cancer patients at King Hussein Cancer Center (KHCC) and American University of Beirut Medical Center (AUBMC) and assessed through patient interviews their cigarette and WPS habits and occupational exposure. Newly diagnosed BC cases aged over 18 years who visited the AUBMC clinics were identified by the research team, and accordingly, the data was collected from January 2017 to January 2019. The inclusion criterion was any adult over 18 years old diagnosed with any type of bladder cancer at AUBMC or KHCC. Pediatric cases were excluded; otherwise, no other exclusions were done.

Following the approval of the Institutional Review Boards (IRB), patients were recruited when they presented to the urology or oncology clinic for a follow-up or when they presented for a urological procedure. A member of the research team directly approached patients after their clinic visit and consented the subjects. An interview was conducted to fill in the study's questionnaire. In addition, some of the Lebanese patients were recruited from a previous study led at AUB for bladder cancer in the Lebanese population [26], and only those who had accepted to be contacted for future studies in that project were approached and consented verbally by the research team. The patient population in this study was compatible with our inclusion criteria of Lebanese BC cases diagnosed between 2012 and 2016 at AUBMC, and a total of 98 patients were recruited from this study. For those patients, the questionnaire was administrated via telephone following an oral consent.

The questionnaire consisted of demographic data, family history of bladder cancer, medical history of the patient, exposure history to the risk factors of bladder cancer, and their social habits. The questionnaire also included an elaborate section on the occupational exposures such as rubber, textile, diesel, coal, gas, blacksmith, glass, and aluminum. Patients were asked to specify the duration of exposure; patients working in any of the mentioned factories or patients who are exposed to duration of either daily or weekly for over one year were considered “frequently.” Social habits that are related to smoking such as coffee and alcohol drinking were also assessed; patients were asked to provide their current number of drinks per day. Smoking habits were meticulously documented; the questionnaire had a detailed section that examines exposure, duration and type of smoking in smokers, ex-smokers, and nonsmokers. In addition, exposure to secondhand smoking was assessed by retrieving data on exposure duration, smoking type, and where the participant was exposed. Patients were considered exposed to secondhand smoking if they are exposed daily or weekly. Heavy smoking is defined as smoking ≥20 cigarettes daily or ≥20 pack years for cigarette smokers and at least smoked once per day for one hour for WPS or other types of smoking.

### 2.2. Statistical Analysis

Numerical variables were summarized by their mean and median. Categorical variables were described by counts and frequencies. In order to compare risk factors between Lebanon and Jordan, we compared the proportions of the smoking habits and exposures. To test the severity of the disease, we categorized the patients in tumor invasiveness and grade and compared their exposures. Cross tabulations in the form of 2 × 2 tables were plotted to compare and detect differences between the two groups in outcome. Since variables on smoking habits, occupational exposure, and tumor were categorical, we opted for cross tabulations to compare them across Lebanon and Jordan. The chi-square test was calculated to detect any significant difference between the two countries, and the Fisher exact test was used where more than 20% of the cells in the cross tabulations have expected frequencies < 5. To compare the mean pack years between the two groups, the Student *t*-test was used. A value of *p* < 0.05 was considered significant in all analyses. All statistical analyses were performed using SPSS v.25.0 statistical package.

### 2.3. Protection of Human Subjects

In order to ensure patients' confidentiality, patients' identifiers were removed and substituted with codes. Only the principal investigator and the research team had access to the names of the patients through password secured files. There were no additional risks, and no direct benefit to the patients enrolled.

This study provides insights on the WPS habits of bladder cancer patients and compares smoking habits and bladder cancer rates and invasiveness between two countries with heavily smoking populations and different occupational exposures to risk factors.

## 3. Results

### 3.1. Patients' Characteristics

A total of 274 bladder cancer patients at the American University of Beirut Medical Center (AUBMC) and 158 bladder cancer patients at the King Hussein Cancer Center (KHCC) were recruited. 235 of the Lebanese patients are male (85.8%), and 39 are female (14.2%). The mean age is 68.9 ± 11.0 SD. More than half of the patients live in the capital Beirut (229 patients; 53.9%) and the remaining either in other regions of Lebanon or outside of Lebanon (29 patients; 10.7%). 249 patients (90.1%) are married. 145 of bladder cancer Jordanian patients are male (91.8%), with a male to female ratio of 11 : 1. The majority (109, 69%) reside in the capital Amman. Only 21 patients (7.7%) have a positive family history of BC in Lebanon compared to 13.9% of Jordanian patients (*p* = 0.045) (Table 1).

### 3.2. Smoking Habits

239 Lebanese patients (87.2%) are current or former smokers. Among them, 193 patients (80.8%) are mainly but not exclusively cigarette smokers; 24 patients (10%) mainly but not exclusively WP smokers; 17 (7.1%) identified themselves as main smokers of both, and 5 (2.1%) mentioned other types of smoking. When asked if they are still smoking, 94 Lebanese patients (34.3%) mentioned being current smokers of any type versus 68 Jordanian patients (43.0%), whereas 145 (46.7%) of Lebanese patients are past smokers, compared to 68 (43.0%) of Jordanian patients are past smokers. Current WP smokers among Lebanese patients are 11 patients (4%) with a mean of 0.9 years, and past WP smokers are 29 patients (10.6%) with a mean of 1.8 years of smoking. Only 12 (4.4%) of Lebanese patients are exclusive WP smokers compared to 3 (1.9%) of Jordanian patients. Among the 239 Lebanese smoking patients, 171 (72.0%) are heavy cigarette smokers and 67 (28.2%) are nonheavy cigarette smokers, and this was statistically different than the Jordanian population that recorded half the Jordanian smokers (67 (49.3%)) as heavy smokers (*p* < 0.001). In Lebanon, 93 patients (34.1%) drink alcohol around 1 glass per day compared to only 22 (13.9%) of participants in Jordan have one glass per day (*p* < 0.001). The coffee consumption was similar in both populations with 242 (88.6%) and 142 (89.9%) for Lebanon and Jordan, respectively. Most Jordanian coffee drinkers consume Turkish coffee (55.7%), similar to the Lebanese patient population 167 (61.3%). The nonsmoker proportion was comparable in both populations (12.8% in Lebanon vs. 13.9% in Jordan) (Table 1).

Most Lebanese patients 193 (70.7%) are secondhand smokers, among them, only 15 (7.8%) are nonsmokers, and the remaining 178 (92.2%) are also smokers (*p* value <0.0001). The types of secondhand smoking are as follows: 131 patients smoking cigarettes (68.2%), 23 patients smoking WPS (12%), and 38 (19.8%) both. In contrast, only 74 (48.6%) of Jordanian patients were exposed to secondhand smoking, and this difference was statistically different than the Lebanese population (*p* < 0.001), from which only 11 (14.9%) are nonsmokers. Types of secondhand smokers in Jordan were 62 (76.2%) cigarettes, 5 (6.2%) WPS, and 14 (17.3%) both. Heavy smokers in the Jordanian smoking population were 67 (49.3%), compared to 69 (50.7%) of nonheavy smokers.

### 3.3. Other Exposures

**Figure 1:**
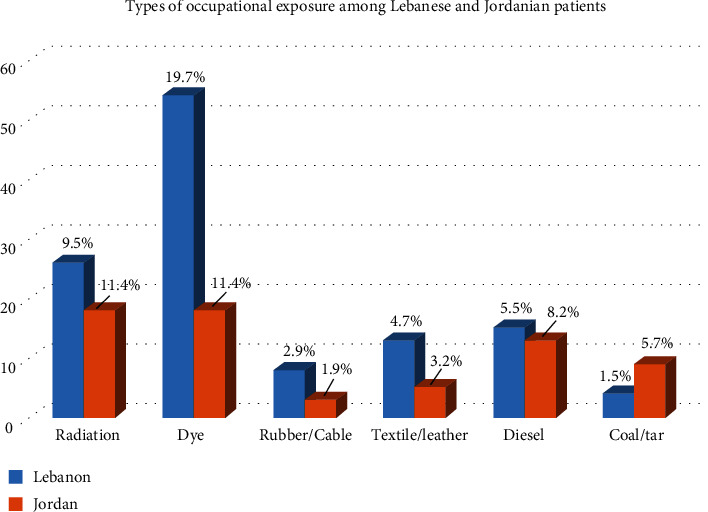
Types of occupational exposure among Lebanese and Jordanian patients. ^∗^*N* and % are from the patient population in each country.

As for the exposure to risk factors of bladder cancer, we included the exposure to radiation, hair dye, rubber, textile, leather, diesel, coal, aluminum, gas, glass, and blacksmith, either occupational or frequently; 94 Lebanese patients (34.3%) reported being exposed to at least one of these, and 180 (65.7%) not being exposed to any of these risk factors. Jordanian patients had greater exposure in general with 76 (48.1%) of them being exposed to at least one occupational exposures; in addition, they had higher exposure to coal (5.7%, *p* < 0.05) and radiation (11.4% vs. 9.5%), but less exposure to dye (11.4% vs. 19.7%, *p* < 0.05) (Figure 1).

### 3.4. Cancer Invasion

Concerning bladder cancer in the Lebanese population, 67 patients (28.5%) presented at an invasive stage, while 168 patients (71.5%) were at a noninvasive stage, 141 (55.3%) with a high-grade tumor and 114 (44.7%) with a low-grade tumor. Interestingly, compared to the Lebanese population, 60.0% of Jordanian patients had invasive bladder cancer at time of diagnosis (*p* < 0.001).

In our study, we found no significant correlations among the 2 groups in the grade and invasiveness of tumor, with the following factors: smoking, secondhand smoking, years of smoking abstinence, alcohol, coffee, family history, place of residence, and exposure to the following risk factors: radiation, hair dye, rubber, textile, leather, diesel, coal, aluminum, gas, and blacksmithing.

## 4. Discussion

The adult smoking is on the rise in Lebanon and estimated at 42.6% (males at 49.4% and females at 35.9%) [18], and the youth smoking is one of the highest worldwide (65.8% for boys and 54.1% for girls) [27], with WP being the major form of smoking in the young generation (33.9%), followed by cigarette smoking (8.6%) [6, 28]. Our Lebanese population showed that exclusive WPS smokers constituted <5% of the total number of smokers. Similarly, smoking in Jordan is increasing, the most recent data on smoking prevalence in men increased in less than a decade from 2011 to 2019 from 54.9% to 66.0% for males and doubled in females from 8% to 17%. WPS smokers constituted around 2% of the total number of smokers in the Jordanian population. The difference in popularity of WPS between the two countries is consistent with the data provided by Jaghbir et al. who sampled a population of 3196 Jordanians and showed that WPS, while associated mostly with the younger generation, represented only 8.6% of smoking habits, and it was most common in the younger generation [19].

Many studies support the fact that smoking promotes the development of bladder cancer, including an analysis performed by Freedman et al. in 2011, who found that former and current smokers had a two- and a fourfold increased risk of bladder cancer, respectively, compared to nonsmokers. At least half of bladder cancer is linked to smoking [29]. A large meta-analysis on smoking and bladder cancer showed that smoking increases the risk of bladder cancer by three-folds compared to nonsmokers [30]. Furthermore, a study conducted in Lebanon on attributable fractions showed that smoking was the cause of 53% and 40%, respectively, of male and female bladder cancer [31].

In a literature review about the effects of WPS on general health, WPS was associated with respiratory diseases, oral cancer, lung cancer, low birth weight, metabolic syndrome, cardiovascular disease, and mental diseases. The existing evidence suggested no association with esophageal cancer, gastric carcinoma, bladder cancer, prostate cancer, hepatitis C infection, periodontal disease, gastroesophageal reflux disease, nasopharyngeal carcinoma, infertility, and mortality [25]. The risk of cancer of WPS is either due to elevated levels of carcinoembryonic antigen (CEA) or to genotoxic and clastogenic components in the WP smoke such as tar and polycyclic aromatic hydrocarbons [10]. In another meta-analysis on cancer risk in WP smokers, the association between WPS and bladder cancer was reported. The summary risk for all studies was 1.25 (95% CI 1.05–1.51) with no evidence of heterogeneity across studies as shown in the measure of heterogeneity (*I*^2^ = 0%) [32].

Bladder cancer is known to be twice as common in men compared to women and in some other studies even 3–4 times more common. According to the National Cancer Registry (NCR), data in Lebanon for 2016 male to female ratio for bladder cancer was 4 : 1 [33]. The main risk factor for bladder cancer is increasing age, as it affects mainly patients aged more than 60-65 years old, due to the cumulative effect of the toxins and the delay between exposure and development of cancer. The mean age was comparable in both countries (68.9 ± 11.0) while the mean age in the Jordanian population is 65.3 ±11.3 years. In our Jordanian population, the ratio of male to female bladder cancer patients is 11 : 1, which is higher than the ratio provided by WHO in 2018 that was around 8 : 1 [34]. This discrepancy is explained by the fact that this number represents the national data whereas cancer patients treated at the King Hussein Cancer Center constitute 35% of the total number of cases in Jordan.

Smoking is a main risk factor, as only 13.2% of both populations are nonsmokers. This result (87.2% of smokers in our Lebanese BC patients' group) exceeds the 46% of male adult smokers and 31% of female adult smokers in the Lebanese general healthy population [35], compared to an estimated 20% of healthy adults who are smokers in the United States and Europe [36]. The Global Adult Tobacco Survey (GATS) found that in 2011, 42.2% of people (55.9% of men and 23.7% of women) aged 15 and above in Jordan smoked tobacco [37]. As with the Lebanese group, our Jordanian group has a higher smoking rate of 86.1%. Remarkably, around one-third of our Lebanese patients and half of our Jordanian patients specified that they are currently still smoking either cigarettes or WP despite their disease, a behavior associated with a worse prognosis. A study proved that current smokers have a greater risk of morbidity following radical cystectomy, and even a short period of preoperative smoking cessation can improve surgical outcomes [38]. The prevalence of smoking among females in Jordan has been rising to 10.9% in 2012, ranking third in Arab countries, while Lebanon ranked first at 24.3% [39].

Smoking duration and intensity are directly related to an increased risk of BC [40]. In our Lebanese patient population, 72.0% are heavy smokers. WP has a social aspect that resulted from special WP cafés and gained popularity among youngsters as initially thought to be a better alternative to cigarettes [6]. We note that even though smoking-free policies exist in both Lebanon and Jordan, but they are not properly enforced in public places, where area separation is not well controlled [21, 41].

Secondhand smoking is also another alarming problem in our Lebanese community. In fact, 70.7% of our Lebanese BC patients described themselves as secondhand smokers. Our statistical analysis shows that secondhand smokers are more likely to also be primary smokers, as 92.2% of the secondhand smokers are also smokers. In contrast, only approximately 46.8% of our Jordanian patients report being exposed to secondhand smoking similar to study done on 500 women showing that around 30% were exposed to secondhand smoking [22]. Secondhand smoking from WP carries a greater risk than from cigarette smoking, as not only does WP contain smoke from the tobacco as well as smoke from the heat source (e.g., charcoal) [42]. To prevent this, more restaurants and outdoor places in Lebanon and Jordan must ban smoking, in order to reduce at least one quarter of the secondhand smokers. Unfortunately, 65.8% of the Lebanese patients mentioned that secondhand smoking is part of their daily life at home, and this should be regulated on an individual basis. The most reported type of secondhand smoking was cigarettes among both groups.

Compared to the data in the UK, where most bladder cancers (75–80%) are noninvasive, we noted a percentage of 28.5% invasive bladder tumors in the Lebanese patients but a percentage of 60% of invasive BC among Jordanian patients. Additionally, 55.3% of the Lebanese population and 53.2% of the Jordanian population had a high-grade tumor. The high percentage of invasive cancer at diagnosis in our Jordanian patients may be due to selection bias, as bladder cancer uncommonly presents at an advanced muscle invasive stage. Specifically, a study done in Jordan on 249 patients at the Jordan University Hospital showed that 82.6% of cases were noninvasive at diagnosis [43].

Occupational exposure is viewed as one of the most important risk factors for BC worldwide [36]. The association between exposure to selected chemical carcinogens and BC is well-established, and it is estimated that 20% to 27% of BCs are linked to occupational exposures in industrialized countries [44, 45]. Lebanon is not an industrial country, yet these exposures are highly prevalent in the country's main cities, especially Beirut and Jounieh. Our data showed that 34.3% and 48.1% of our Lebanese and Jordanian populations, respectively, reported a frequent exposure to hair dye, rubber, textile, leather, diesel, coal, aluminum, gas, or blacksmithing. These are high percentages in comparison to other countries.

Another risk factor in Lebanon would be the environmental pollution, particularly of water and air, suspected to increase the risk of BC. The pollution of drinking water is by chlorination, exposure to trihalomethanes, pesticides, and heavy metals including arsenic [46, 47]. Air pollution of the petrochemical type is mainly secondary to the diesel-fueled electric generators present in most Lebanese areas and to the high traffic load [13]. Considering that some cities like Beirut are much more polluted, no significant correlation was found between the residence place and the invasion/grade of the tumor. Another study assessed particle oxidative potential in the greater Beirut area [10]. Metals and trace elements of vehicular origin displayed increased levels at the roadway, and elements with reactive oxygen species (ROS) activity (of particular concern) were 1.5–2.8 times greater near the freeway, constituting an additional health risk. Compared to other urban cities like Los Angeles, the particulate matter-induced ROS activity is 2.3-fold greater in Beirut and 3.1-fold the activity in the heavily polluted Lahore, Pakistan. Another study suggested that Beirut experiences a high particulate matter that exceeds the WHO annual averages, being at the peak in fall and summer as a result of low precipitations, the increase of dust storm activities, and the increase in traffic density [48].

According to the WHO, the air quality in Jordan is considered moderately unsafe. The most recent data indicates the country's annual mean concentration of particulate matter (PM2.5) to be 33 *μ*g/m^3^, exceeding the recommended maximum of 10 *μ*g/m^3^ [49]. Lebanon by comparison has a PM2.5 of 31 *μ*g/m^3^ [50]. Both countries therefore exceed the safety limits established by WHO, and interventions need to be implemented to provide safer air and reduce risk of disease.

There are currently no guidelines that recommend screening for bladder cancer in Western countries [51]. Screening for bladder cancer in high-risk populations is controversial [52], with a need to further study its benefit in Lebanon and Jordan [53].

## 5. Limitations of the Study

The low percentage of exclusive WP smokers among our BC patients limits the possibility to draw conclusions from the effect of WPS on BC and other cancers in the future. Finding correlations between the invasion/grade of the tumors with BC risk factors was also limited. The data was helpful in describing and exploring smoking behaviors among BC patients on patients in two Middle Eastern countries. The high percentage of muscle invasive BC in our Jordanian patients is likely related to selection bias, and further data collection from other centers is needed.

## 6. Conclusion

Bladder cancer is on the rise in the Middle East, especially in Lebanon with it being the second most commonly reported cancer among males and fourth most common in Jordan. Smoking is a major risk factor for bladder cancer, and both cigarette and WPS are rising in popularity in Jordan and Lebanon. Secondhand smoking is also a poorly controlled risk that requires more strict smoking policies to prevent exposure in public settings. More data is needed regarding WPS and bladder cancer association especially given the rise in popularity of hookah smoking and its use in both public and private setting.

Further studies should look into bladder cancer screening and whether they offer a significant clinical benefit in high-risk populations. More studies need to also be conducted in Jordan regarding bladder cancer invasiveness, as our sample patient population in Jordan presented with a moderately higher percentage of invasive bladder cancer versus worldwide data.

## Figures and Tables

**Table 1:** Smoking, social habits, and tumor characteristics of our Lebanese and Jordanian populations. WPS: waterpipe smoking.

Variable	Lebanon		Jordan		*p* value
	*N* ^∗^	%^∗^	*N*	%	
Smoker					0.769
Yes	239	87.2	136	86.1	
No	35	12.8	22	13.9	
Smoking type					0.058^∗∗^
Cigarettes	193	80.8	112	83.0	
WPS	24	10	7	5.2	
Both	17	7.1	16	11.9	
Other	5	2.1	0	0	
Heavy vs. nonheavy					<0.001
Heavy	171	72	67	49.3	
Nonheavy	67	28.0	69	50.7	
Smoking status					0.127
Smokers	94	34.3	68	43.0	
Ex-smokers	145	53.0	68	43.0	
Nonsmokers	35	12.7	22	13.9	
Pack year	47.6		31.5		<0.001
Secondhand smoking					<0.001
Yes	193	70.7	74	46.8	
No	80	29.3	84	53.2	
Alcohol					<0.001
Yes	93	34.1	20	12.7	
No	180	65.9	138	87.3	
Family history					0.045
Yes	21	7.7	22	13.9	
No	253	92.3	136	86.1	
Coffee					0.750
Yes	242	88.6	142	89.9	
No	31	11.4	16	10.1	
Occupational exposures (1≥)					0.004
Yes	94	34.3	76	48.1	
No	180	65.7	82	51.9	
Tumor invasion					<0.001
Yes	67	28.5	84	60	
No	168	71.5	56	40	
Missing					
Tumor grade					0.684
High	141	55.3	33	53.2	
Low	114	44.7	29	46.8	
Missing			96		

^∗^ *N* and % are from the patient population in each country. ^∗∗^Fisher exact test.

## Data Availability

Data related to Lebanon are found in the American University of Beirut Medical Center, Division of Hematology and Oncology. Data related to Jordan are found in the King Hussein Cancer Center (KHCC).
